# COVID‐19's ambiguous parcel: Agency, dignity, and claims to a rightful share during food parcel distribution in lockdown South Africa

**DOI:** 10.1002/sea2.12224

**Published:** 2021-08-20

**Authors:** Magnus Godvik Ekeland

**Affiliations:** ^1^ Anthropology and Development Studies Radboud University 6525 XZ Nijmegen the Netherlands

**Keywords:** COVID‐19, Agency, Dignity, Rightful Share, Social Assistance

## Abstract

During the South African lockdown, food relief was largely delivered by civil society, after the government failed to honor its pledge to provide for the population. By taking a local food parcel initiative in a small rural township as an ethnographic case study, this article examines why the attempt to mimic government food distribution generated dissatisfaction and tension in the targeted community. At the heart of this argument is the way the local parcel project created ambiguous anticipation regarding the consequences for residents' experience of dignity, as it undermined an established binary understanding of economic dependency. This understanding clearly distinguishes between relationships within the community and those with the government. Proximity and intimacy increased the risk of public humiliation arising from indebtedness, while dependence on the government did not inevitably generate the same anxiety. Agency is identified as a key signifier to understand strategies for upholding dignity in the different relationships. This article examines different stakeholders' spontaneous and creative attempts to control the relational outcome of the distribution. It discusses how narratives about the distribution as a rightful share to the community's wealth emerged among potential recipients in an attempt to negate any notion of personalized reciprocity following the distribution.

It was a gray, chilly morning in late April on the day the long‐anticipated food parcels were due to be delivered in Alexandra, a small rural town in the Eastern Cape in South Africa. This was the culmination of weeks of planning by an assembly of local stakeholders popularly known as the COVID‐19 committee, which had decided to act after food relief from the government had failed to materialize. A rustic church hall, which twice a year was the scene of a cattle auction, served as the collection point for the parcel items—flour, sugar, milk, salt—all sorted in equal number and placed in translucent plastic bags that had been scattered around on the floor, with roughly a meter between each bag. Two community leaders from the predominantly Coloured township were busy arranging the bags. Morgan, a burly, late middle‐aged man with a firm stare and grip, and Simon, an older man with a composure that easily moved between authority and lighthearted joking, both had been part of the committee since its inception the week before the commencement of the lockdown. Now they looked forward to the end. While I assisted them with the arrangements, others began arriving, mainly middle‐aged men, clustered together by the wall, waiting in silence. Simon explained how they had asked some of the men who would receive parcels to stop by to collect their bags, to reduce the number of houses the committee would need to visit later that day. “Everyone, walk to one parcel, and stand beside it” (fieldnotes, April 27, 2020), Morgan called out in a stern voice. “We're going to take some photos, to document who are receiving parcels.” The silent crowd walked toward the parcels, guided by Simon and other committee members. While photos were snapped, Simon turned to me smiling, telling me how brilliant the idea was to visually document who got a parcel.^1^ Afterward, the parcel receivers hurried out. With the men well on their way, we loaded the remaining parcels onto the assembled *bakkies* (pickup trucks); some headed toward the Xhosa township, while I joined the ones heading toward the Coloured township. During the next hour, we drove up and down the streets. By each house, recipients had to sign their name on a piece of paper, then pose for a photo while holding their parcel. Not everyone was excited about being photographed. One of my study participants, Mary, in her mid‐thirties, protested, pointing out that she was wearing a nightgown. Her protests fell on deaf ears, and she ended up posing reluctantly, not bothering to smile. One of the drivers commented that if everyone who requested this would be allowed to change, it would take the whole day. At the end of the day, 132 parcels had been delivered to an equal number of households.

During his speech announcing the upcoming national lockdown, President Cyril Ramaphosa reassured the population that his administration would provide relief for those whose livelihoods would be affected by the national lockdown (Ramaphosa 2020). Anticipation was particularly geared toward the delivery of food parcels. While studies of social welfare in South Africa tend to concentrate on the system of means‐tested social grants (Hanlon, Barrientos, and Hulme 2010; Ferguson 2015; Neves et al. 2009; Surender et al. 2010), food relief schemes have an equally long history in South Africa, being run either by the government or by civil society (Seekings 2020). Yet these existing institutions were overwhelmed by the scale of the crisis. A contemporary piece by the *Daily Maverick* accentuated some of the problems hampering the government's response, including lack of capacity for delivery, lack of data on potential recipients, theft, and poor coordination with private initiatives (Davis 2020). Given these problems, a major mobilization was undertaken by local governments together with civil society, including nongovernmental organizations (NGOs) and local initiatives (Seekings 2020, 22–27). A consequence was that distribution varied not only between but within provinces, depending on factors such as the number of active NGOs, local beneficiaries, or the capability of municipalities. To give just one example in a smaller township about forty‐five minutes from Alexandra, a local NGO together with local residents managed to organize several rounds of distribution, providing a parcel for almost every household. No NGOs of that kind were operating in Alexandra, and apart from farmers, few potential donors were ready to help.

In this article, I will discuss the local initiative to distribute food parcels from its inception until the aftermath of the distribution. The perspectives of both the organizers and the potential recipients will be discussed. As I will argue, the local food parcel project became a source of much ambiguity in the community and possibly created more tension, because there was great concern over how this would impact notions of dignity. Key to grasping these sentiments is how residents in the township distinguish between two sets of relationships: those concerning family and neighbors and those toward the state and a range of financial institutions. Notions about agency and how to preserve dignity are different between these relationships, together with the potential risk of becoming indebted and, thus, humiliated. The relationships the parcel project was imagined to create did not fit neatly into this existing binary categorization. Residents responded by improvising strategies for reasserting agency and dignity in the face of a confusing event, claiming the right to define themselves through relationships that would be created by this distribution.

## Research methods

This article is based on ethnographic fieldwork conducted from September 2019 to October 2020. The names of study participants and of the town itself have been changed. I began fieldwork in Alexandra in late September. In December, I moved to the outskirts of town, staying until May, when I relocated to a township house for the remainder of my stay. My study participants were primarily between twenty and forty‐five years of age, with most being unemployed or having casual employment. My days were generally spent in the township, from morning to nightfall. Although I visited both townships regularly, my main focus was confined to residents of the Coloured township, who also are the subjects of this article. The restrictions on movement imposed as part of the national lockdown created some obstacles for conducting participant observation. During the first month, I attempted to meet daily with my study participants at the stores, which we could visit between 10:00 a.m. and 6:00 p.m. every day, and to keep in touch over WhatsApp or landlines.

After joining the local COVID‐19 committee, there were more opportunities for visiting the township as part of the committee's work to disseminate information. As time went by, I would also make shorter trips on my own, in agreement with my study participants and respecting regulations concerning social distancing. After joining the committee, my wider network of study participants began questioning me about the meetings. Likewise, members of the committee, particularly Simon and Morgan, encouraged me to share my impressions from the meetings widely but also to refer anyone with questions directly to the committee members. While being perceived as a suspicious character is a common experience among anthropologists, similar to Clifford Geertz's (1972, 1–5) classic example in the introduction to his article on “Deep Play,” Simon was convinced that residents would consider my renderings of the committee meetings more reliable than those given by himself or other local committee members. He attributed this to the fact that because I was not South African and was relatively new to the community, I would not be seen as a stakeholder. However, while others largely agreed, some highlighted how, as a foreigner, I was blind to important subtleties in manner of speech and action. Thus I cannot assess if my study participants' perceptions of the distribution were significantly influenced by what I divulged from meetings. I have discussed my interpretations of these events with some key study participants over WhatsApp.

## Rural townships

Alexandra is one of those old, rural railway towns where the train no longer stops. The town proper is small, with the vast majority staying in the adjacent townships. Thirty years after apartheid was abolished, residential patterns had not undergone much change, with the population in the two major townships considering themselves as Coloured and ama Xhosa, respectively. Unemployment runs high, despite a number of factories situated close to town creating some opportunities. The surrounding area witnessed a large expansion of game farms in recent decades, and today, a substantial number of residents from the townships find employment on these farms, although it is questionable whether the game farms have significantly boosted job creation in the region (Spierenburg and Brooks 2014). At the beginning of the lockdown, the factories were allowed to continue normal operations, while the largest game farms continued to pay partial salaries to their permanent staff, most of whom were sent home indeterminately.

However, like so many deprived communities across the continent, residents rely, to a varying degree, on participation in reciprocal networks of exchange, where negotiations about material assistance take place under the guise of local norms for appropriate conduct (du Toit and Neves 2013; Ferguson 2015; Nyamnjoh 2015). James Ferguson (2015, 94–102) has presented *distributive labor* as an umbrella term for the multitude of creative and improvised strategies people use to get by. There was little lenience; even close family members expected loans to be paid in due time, and conflicts over money were common. As Warren, in his mid‐twenties and unemployed, explained, it was pivotal to nurture reciprocal relationships with several individuals. That way, he avoided having to take a single big loan that he would struggle to repay (interview, September 10, 2020).

As he told me, being indebted to someone would leave him publicly humiliated, and this frightened him to the extent that he would rather underborrow. Fiona Ross (2009) has highlighted how avoiding humiliation together with persevering *ordentlikheid* (respectability/dignity)^2^ underpin all types of distributive labor among Coloured residents in a deprived part of the Cape Flats. While Alexandra's residents lived under much more favorable conditions, the norms for negotiating assistance were similar. For Warren, preserving dignity was closely tied to his agency, because he strove for freedom of choice regarding whom he could approach. The fewer people he potentially could ask, the greater became the risk of being forced into accepting unfavorable terms with heightened risk of indebtedness. Ross's (2015, 105) distinction between borrowing and being indebted is useful for understanding this complexity; borrowing assumes equality between two parties, whereas indebtedness is associated with hierarchy and the risk of humiliation. Warren's fear was Bradley's reality. A former army officer, now unemployed, he lived with his four children, partly relying on borrowing from his married sister. However, after discovering how his brother‐in‐law had been gossiping about how Bradley was living off *his* salary, he stopped borrowing from her. He confessed how the humiliation of being publicly ridiculed had made him reluctant to borrow in general, although he now struggled to cover the cost of electricity and food (interview, December 19, 2019). During lockdown, several of my study participants dreaded the possibility of indebtedness to a neighbor or family member more than potentially going hungry.

## Two forms of dependency

In the wake of the president's speech, residents were informed that they could apply for a food parcel provided by the South African Social Security Agency (SASSA) by filling out their personal details on a list at the police station.^3^ Mary, who once told me she had neither seen nor smelled a parcel before, despite applying for one in the past, admitted to cradling a sliver of hope this time. A food parcel would save her from a lot of stress. Her recent attempt to take out a bank loan to cover the anticipated expenses ahead had been declined, and while she was busy “making a plan” (fieldnotes, April 2, 2020), she hoped to avoid borrowing from family or neighbors. Although dissatisfaction with official service delivery was relatively common, people directed their anger mainly toward the local municipality.

Corruption and mismanagement were frequently cited as reasons for the pitiful state of roads and the irregular access to tap water. On the other hand, they were much easier on government departments and agencies like SASSA. Application processes were described as unnecessarily tedious, but the procedures themselves were mostly deemed fair. Profoundly, worries about dignity and risk of humiliation never entered the conversation whenever we talked about SASSA's parcels. To understand why, it is fundamental to grasp how residents distinguished between interpersonal relationships in the township and relationships with a wide variety of institutions, including the government and different financial institutions. This binary categorization of relationships has implications for the experience of agency, dignity, and the perceived risk of humiliation. The observation that receiving a grant did not beget an experience of being indebted, even though recipients recognized themselves as dependents of the government, serves as an illustration. Ross (2015, 105) has made similar observations, noting how indebtedness and dependency on others in the community are weighed differently compared to being dependent on the state, due to impersonality and lack of proximity. This concurs with wider scholarship that has postulated how the experienced unequal relationships of dependency and indebtedness are shaped by factors like social proximity, familiarity, and kin (Ferguson 2015; Graeber 2011; Gregory 1982; Martin and Yanagisako 2020). If we factor in relationships with financial institutions, specifically for money lending, this becomes more complex.

According to Warren's friend Mark, negotiating loans from friends or family came with an expectation that you would explain how you intended to spend the money (interview, January 2, 2020). Even if they agreed to grant him a loan, they would keep an eye on his activities, particularly if he was late with his installments. It was difficult to keep a low profile in such a small community, he lamented. The loan sharks he frequented were discreet, although they held on to his identification card as collateral. Although accepting the terms of a loan shark involved temporally suspending his agency, he avoided becoming indebted to friends or family. Erik Bähre (2007) has discussed how saving clubs, popularly known as *stokvels*, despite being created as a strategy to counter economic depredation and protect dignity, were plagued by mistrust and, in some instances, ended up generating more tensions. Mark admitted feeling embarrassed about relying on loan sharks, but it allowed him to hide this dependency from the wider community. He could keep his head high when leaving the house.

## The COVID‐19 committee

Already before the country officially went into lockdown, a local COVID‐19 committee had been formed to discuss how to mitigate the negative effects of such an eventuality. The committee was made up of representatives of the municipality: local business owners, farmers, and community leaders; the latter constituted the majority. During the early days, the most pressing issue at hand was to inform residents about recommended precautions for avoiding infection, together with clarifying lockdown regulations. Rumors circulated over WhatsApp and other online platforms, with chain messages framed as if they came from the government despite official counterefforts (Anciano et al. 2020a). Reclaiming the information monopoly was a key objective, and the committee quickly decided to organize door‐to‐door canvassing in the two townships, which was the time I was invited to join the committee. During the canvassing, I accompanied Simon, Morgan, and a handful of other community leaders. Simon had brought along a piece of paper to note down the names of those to whom we spoke. I was curious if this was to register the number of people in each house. Simon sent me a puzzled look. “No,” he told me, and he explained that it was just to document that they in fact had spoken with everyone staying in the township (fieldnotes, April 4, 2020). It was only later that the significance of this action, and its connection to transparency, would become clear.

## Performing transparency

Neither Simon nor Morgan was officially employed in the state apparatus; instead, they were chosen as representatives from their townships/communities. Municipal officials and businesses/contractors were supposed to consult with the community leaders before making any decisions impacting the community. Given how the municipality was the subject of countless rumors about corruption, together with the widely held belief that intentions revealed themselves through actions, community leaders were closely monitored. Well aware of this suspicion, community leaders like Morgan and Simon put great effort into being as transparent as possible. Foremost were attempts to organize community meetings regularly, where they summarized their activities in detail. Moreover, Morgan liked to stress how important it was to be available for anyone seeking his assistance or having questions. I have termed their actions for the purposes of transparency *performing transparency*. These efforts were a strategy for both reasserting their authority and protecting their own dignity. Failure to perform transparency could easily lead to the humiliating reality of being branded a crook. Although neither Morgan nor Simon explicitly made a point of it, the fact that both were unemployed and lived relatively modestly probably gave them some leverage. Furthermore, neither socialized much with other residents. I hardly knew anyone with whom they had intimate relationships apart from their nuclear families.

The lockdown complicated matters. It would be a long time until the next community meeting, and many were mystified about what was actually being discussed in the committee meetings. A tragic‐comic effect of this occurred in relation to the expected food parcels, about which many of those with whom we spoke during the canvassing inquired. Simon and Morgan had helped with informing residents about the existence of the SASSA list before the lockdown, but now they replied that they had received no updates about the parcels from SASSA at all. Nobody questioned their response outright, but other study participants later told me how many had begun suspecting the committee was holding back information, for how could they not know anything when they were the link to the government?

## Mysterious parcels

Two weeks went by after the canvassing without any updates concerning the timing of the arrival of the SASSA parcels. In response, the committee had set to work on a local food parcel project, yet the organization was still in its infancy. I was thus baffled upon receiving a text from Lucy (fieldnotes, April 6, 2020), a close study participant, inquiring about the parcels that had just been distributed to residents in one of the informal settlements. My first inclination was to dismiss it as just another rumor, but she replied that she had heard it directly from one of the recipients. Moreover, it had been a representative from the municipality, along with Bob, a local businessperson‐cum‐community leader, who distributed the parcels. He usually participated actively in the committee, but he had not mentioned anything about distributing parcels. Because there were some days until the next committee meeting, Lucy agreed to set up a meeting the following day with someone she knew had received a parcel.

We met by one of the shops in town, given the restrictions on movement. The lady I was meeting, Janine, had stayed for a couple years in the informal area together with her father. Both were unemployed, her father having suffered an accident at work that left him partially paralyzed, and Janine was unable to apply for a job because she did not have an identification card. They got by through types of distributive labor, particularly making tiny loans or doing piece jobs, such as cleaning or simple repairs, in exchange for money or food (cf. Ferguson 2015, 94–102; Ross 2009, 123–29). Opportunities for pursuing these strategies had become more limited for father and daughter during lockdown. Intriguingly, this was not because of monitoring of curfew by the police but rather because they had no kin in town. Boundaries of reciprocity had retracted during lockdown, with many only lending money to family or close friends. Morgan had mentioned this reality in a previous meeting, highlighting how the situation was only really dire for those who suffered from being both unemployed and having no family in the town. His suggestion to prioritize these people in the forthcoming distribution received unanimous support.

“So the parcel was a blessing?” I asked Janine while she was lighting a cigarette (interview, April 7, 2020). “No, it was humiliating,” she replied quickly, before inhaling deeply from the cigarette several times. The parcel had barely contained anything at all; it would barely last a week, she told me, citing the tiny bags of meal, potatoes, and sugar as examples. She was further annoyed because she did not understand on behalf of whom the parcels were being distributed or why she got a parcel while her next‐door neighbor did not.

Expecting that someone could get by on so little was humiliating enough, but it was not fair to provide parcels only for a select few. Whoever it came from, she had not asked. Janine emphasized how quick the distribution had been. It was over before she had comprehended what had taken place. She was confused, and I likely added to her frustration when I pointed out that to my knowledge, this was not approved by the committee. The residents in the informal settlement were often depicted as Alexandra's own “wretched of the earth,” spoken of with a blend of sympathy and aversion. Housing conditions were poor, and they shared a single water tap.

However, the population was heterogeneous, and turnover was frequent because many moved there temporarily following conflicts with family. A shared trait was the weak social ties to the township proper. I visited the informal settlement a couple weeks after meeting with Janine to talk with her neighbors about the distribution. Together with Janine, I sat down with a large group, who were roasting bread over an improvised griller. One lady was quite agitated about Bob's distribution, citing the unfairness of only providing parcels for some, while a couple complained about how they were not properly informed who was behind the distribution (fieldnotes, April 25, 2020). An old man wondered aloud if this would interfere with SASSA's distribution. “I watched on TV the big parcels they gave out in Johannesburg,” he said, shifting the conversation to the contents of the parcels. SASSA had delivered parcels there in the past, so Bob's parcels were now compared with the contents of SASSA's, together with those seen on television or social media. It is possible recipients feared they would be perceived as indebted to Bob, without having any chance to reciprocate. Everyone denied having any relationship with Bob. Critical here is how the distribution suspended agency. Janine and her neighbors frequently visited households in the townships requesting abysmal loans or offering to do piece jobs. Yet they were always the ones initiating the negotiations over assistance.

Naturally, the distribution became a topic at the following committee meeting. Bob spoke first, calmly, yet with eyes flickering more than usual. He told us how he, together with Daniel from the municipality, spontaneously had decided that something had to be done for residents in the informal settlements (fieldnotes, April 8, 2020). SASSA bureaucrats were taking their sweet time while people were starving. They had persuaded some of the local shop owners to sponsor them with a few household items. It had been enough to make a dozen parcels, he said. He acknowledged that it was a bit shortsighted in retrospect but emphasized that they acted on the grounds that something had to be done fast. The rest of the committee had remained silent, but now several members voiced their disapproval, particularly at how Bob and Daniel had failed to clear the parcels with the committee beforehand. Both Morgan and Simon criticized the endeavor as problematic, given all the time the committee had spent trying to avoid confusion about the food parcels. After a handful of critical questions, the meeting moved on to the next point on the agenda.

In the wake of the meeting, I discussed the incident with Morgan and Simon. Simon felt dejected, telling me how people now probably speculated about Bob's motives for distributing the parcels to only selected households (fieldnotes, April 8, 2020). Those who knew Bob insisted that he had not had any hidden intentions. His cousin Mendel told me how Bob was only looking to help people, citing Bob's previous involvement with school and sport projects (interview, August 8, 2020). Among those who had no relationship to Bob, more malign rumors circulated. The first, which I picked up only days later and which would reappear in different shapes after the official distribution, claimed that parcels had been meager because items had been removed beforehand. During a talk at the shop with Lucy and Mary a couple days after the committee meeting, Lucy insisted that I had to remember to count every arriving parcel, while Mary shook her head, saying it would be easy for someone to remove a parcel without anyone noticing (fieldnotes, April 22, 2020).

When the local initiative became known, it was followed by rumors that the planned parcels in reality might be SASSA's and had been confiscated by the committee. What was profound about these rumors was that barely anyone accused the government of engaging in parcel fraud. Suspicions were almost exclusively directed at the local municipality and the committee. Boris Gershman's (2016, 3) impressive documentation of the correlation between trust and witchcraft beliefs across eighteen sub‐Saharan African countries offers some support for this assumption, as in those areas where Gershman found both interpersonal trust and trust in local institutions to be low, his respondents displayed more trust in the “larger government.” This might support an assumption about pervasive mistrust toward interpersonal relations and the municipality, which was somehow being balanced by preserving trust in the government. Another dimension is how the risk of public humiliation is shaped by proximity, which makes the risk significantly higher when interacting with a representative of the municipality, who may very well live in the township, compared with a city‐dwelling government representative.

## Ambiguous anticipation

“You know how our people are; they never want to listen to us” (fieldnotes, April 15, 2020). The setting was the weekly meeting of the committee one and a half weeks after the first distribution of parcels, and participants were voicing their frustrations about how many residents seemingly ignored the new lockdown rules. About twenty‐eight people had turned up, only a few of them women and hardly anyone younger than thirty years. “It's not just our people, even persons of authority show a lack of understanding of these regulations,” sighed the chairperson, rubbing her temple. She was greeted by silence. Morgan had confided that he regularly visited his old mother, and several others on the committee later admitted to having violated the curfew. “But we have to keep in mind that there has been no water in town”—it was Paul, a heavily built contractor, who relieved us from the uncomfortable silence. Morgan nodded and spoke up. “Yes, can anything be done with the water? There are so many coming to my house asking for water these days. I had to put a padlock on my tap to keep them from taking at night!” Morgan had told me earlier how he never turned away anyone who came to his house for water, as long as the person asked. This was met with several exclamations of agreement.

The roles had now suddenly been reversed, and it was the chairperson who was faced with the uncomfortable reality of the municipality's failure to fix the water supply. She quickly changed the subject, informing us that she had finally heard from SASSA. “SASSA informed me that they would provide twenty‐nine parcels, but I was told five hundred put their names down on their list.” She allowed for a moment of silence so the message could sink in. “How far are we with the parcels from the local businesses and farmers?” A farmer signaled that he wanted to reply: “Ninety percent of those we have been in contact with have been positive. We initially wanted to wait for SASSA to distribute theirs, so there would be no confusion about where the parcels are coming from.” He went on to inform us that slightly more than one hundred parcels should be feasible and that he agreed that it was futile to continue to wait for SASSA. The next step was to begin compiling a list of eligible residents.

In compiling the list, the committee had to decide on the selection criteria: Should they aim for equality or equity? A suggestion to give parcels to individuals but restrict parcels to one per household was agreed upon. However, this meant that some parcels would be distributed to households where one or more members had a stable income, while households where several were unemployed would have to share one. Curiously, there were hardly any references to the earlier distribution. Even Bob, who participated actively in the discussion, refrained from drawing explicitly on his distribution attempt. Yet it was difficult to imagine topics such as the size of the parcels becoming a significant discussion point without considering the dissatisfaction reported by the first parcel recipients. The committee settled on well‐stocked ones, even if it would mean fewer parcels overall. Likewise, community leaders took care informing residents about the upcoming distribution, emphasizing how the project was organized by the local committee and highlighting that items for the parcels were donated by *spaza* (small shop) owners and farmers.

Although local businesses were providing the funding, community leaders were the ones with the real decision‐making power, as the committee had agreed that they were best suited to compile a new list, given their intimate knowledge about who was struggling. Exactly how community leaders would select individuals for the list was not entirely clear. Morgan was ambiguous about using SASSA's list. Relying on it could potentially create rumors about the committee actually distributing SASSA's parcels. Furthermore, they were not sure everyone on the list really was eligible because someone might have hoped to obtain a parcel for which the individual did not qualify. During the planning, it became apparent that the distribution process would mimic distributions carried out by the government. Why they emphasized that it was on behalf of the committee and who exactly constituted the committee were still puzzles to many residents.

Gradually, however, a pattern emerged where people began referring to the anticipated distribution in a language resembling the discourse of the “rightful share,” in the sense of people asserting an entitlement to a portion of the nation's accumulated wealth (Ferguson 2015, 168–89). Over the last decade, such claims have increasingly been popularized (Barchiesi 2007; Ferguson 2015; Widlok 2017,152–57). In Alexandra, these narratives were, curiously perhaps, not tied to the use of land or national resources, as in other parts of the country. For example, Erin Torkelson (2020) writes about farmworkers drawing on their history of toiling with the soil to assert themselves as net creditors to the nation's wealth. An absence of this discourse in Alexandra was particularly surprising because many residents were descendants of farmworkers and a substantial number of them were employed on the surrounding (game) farms (Connor 2005). Warren, Mary, and Lucy all had parents or grandparents who had lived at farms, but they had grown up in the township and were more oriented toward the city.

Instead, claims were directed almost entirely toward businesses in town, particularly the spazas, which were run exclusively by immigrants. In these narratives, store owners were presented as profiting on the community during the crisis, a recent price hike being cited as proof. Lucy sarcastically remarked how store owners cared more for profits than for the well‐being of the community (fieldnotes, April 20, 2020). Asserting a claim to a rightful share also contained a leveling dimension, because it took aim at those perceived as entertaining a close relationship with either store owners or community leaders, who were suspected of surreptitiously attempting to use their relationships to reserve a parcel. Although these narratives resembled the discourse adopted by the committee, where business owners and farmers were spoken of as members of the community with corresponding responsibilities, the committee framed the upcoming distribution as a gift, not a share.

## Distribution as a share

Reimagining the distribution as a type of share rather than a gift was just one of the strategies used to preserve dignity in the face of the distribution. Among my wider network of study participants, anticipations were not high about the distribution. It was as if many were gearing themselves up for disappointment, even Mary and Lucy, who were on the list. They were practically sure about their inclusion because they had discussed the matter with Morgan after spotting him by a spaza. “We saw Morgan by the shop, and I told Lucy we had to tell him about our situation, or else he can claim he didn't know we needed parcels,” Mary told me (fieldnotes, April 23, 2020). Janine's narrative echoed in the words of Lucy and Mary. They sought to avoid what Janine had experienced, and by taking initiative to discuss the matter with Morgan, they reasserted their agency. Because they, too, were committed to the narratives about receiving a rightful share, they could project the negotiation between them and Morgan as confirming the imagined equality between residents.

These strategies arose spontaneously yet resemble a broader set of creative improvisations to which people in poor and vulnerable communities turn when they feel threatened by the imposition of authority (Scott 1985). In Alexandra, community leaders were suspected of attempting to accumulate more power, in which the compilation of the beneficiary list features as the most striking example. When food parcels had been delivered in the past, social workers had been tasked with assisting applicants, but they were not living in town, and to my knowledge, none visited during the first months of the lockdown.

## Soup kitchen

Two rounds of distribution had initially been planned, but when the committee gathered again a couple days after the distribution outlined in the beginning, they decided it was more feasible to organize soup kitchens for the children. This had been on the agenda earlier, particularly because children were missing out on hot meals after schools closed (Spaull and van der Berg 2020). This decision was strengthened by talks that some of the larger game farms would distribute parcels. It was agreed that the organization of the soup kitchens would be left to the community leaders from the respective townships. Morgan led the first meeting in the Coloured township and opened by suggesting they aim for three days a week and dish up until the prepared pot was empty (fieldnotes, April 29, 2020). The attendees, other community leaders together with some ladies who had been involved with an earlier church‐led soup kitchen, agreed. “This is how we do it here,” Morgan finished, addressing me. “Our children play together regardless of background. Of course, some need this food more than others, but a child does not understand why only some get and not everyone,” one of the ladies added with a tender smile.

With the distribution of food parcels fresh in mind, I assumed the preference for equality over equity was shaped by a desire to avoid being seen as favoring children of kin or friends, but no one ever brought this up. Instead, they emphasized how they were modeling the organization on earlier community‐led soup kitchens. These had never turned children away as long as there was food left. Residents were generally positive about the kitchen, but they made it clear that they expected it to operate like the former kitchens, indicating that equality should take precedence over equity. Similar to negotiations of loans, efforts were by all parties geared toward upholding an imagined equality. The reactions to Bob's parcels illustrated the tensions that might erupt when existing social inequalities were publicly illuminated.

## Agency, dignity, and the share

A best‐case scenario was to run soup kitchens a couple of days a week, while parcels from the farms, together with the long‐awaited SASSA parcels, would satisfy those who missed out on the first round. Unfortunately, when thirty parcels from SASSA arrived in mid‐May, many instantly became convinced that someone had removed the majority of the parcels beforehand. Residents found it hard to believe that the government had delivered so few. Although the committee was not directly involved in the distribution of these parcels, it still faced the bulk of the suspicion. Although an impressive number of residents were covered by the local parcel project, widespread ambiguity about the distribution remained, and despite the efforts of the committee, I met residents who believed the parcels came from SASSA. A bigger concern was how the aftermath was plagued by reactions similar to those that had haunted Bob's distribution. A peculiar response was the vocal complaints about the items in the parcels, which bordered on ridicule. Such behavior is common in communities where sharing is institutionalized (Widlok 2013, 2017). As Thomas Widlok (2013, 18) has highlighted in his studies of sharing, the act is both preceded and followed by gossip, and those providing the share are ridiculed. The notion of the rightful share might illuminate how residents solved the ambiguity of the distribution. Concerns about being indebted and humiliated evaporated quickly following the distribution.

Instead, the talk concentrated on entitlement to the parcels and gossip about parcels being removed. Generally, these narratives rejected any element of reciprocity; even being overly grateful was shunned. Ambiguity about how to categorize the type of relationship created by this distribution drove residents to dismiss the existing binary categories, imagining a new relationship where reciprocity was negated. Simultaneously, rumors and suspicion created more tension in the community and delivered a hard blow to the legitimacy of community leaders, who felt the full weight of these rumors. Simon and Morgan were both so exhausted from being the target of persistent suspicion that they contemplated withdrawing from their positions. According to Nancy Scheper‐Hughes (1996, 9), malicious rumor can also “signify a sense of alarm, warning people in the community that their bodies, their lives, and those of their children may be in danger.” It is likely these rumors had a similar function in the township, urging people to become more vigilant in monitoring the actions of community leaders particularly of attempting to craft new hierarchical relationships through the distribution.

The literature on wealth‐in‐people, concerning how a person is ascribed value, is helpful for understanding these concerns, because it directs attention toward how exchange can be a means to establish rights in another person (Kusimba 2020, 169–71). As Sibel Kusimba (2020, 170) argues, in relation to the introduction of money in rural Liberia, introducing novel objects of exchange can create opportunities for obtaining rights in others. This illuminates why residents suspected that parcels were a ploy to cajole them into relationships where they could become indebted. However, as the literature on wealth‐in‐people postulates, being dependent does not necessarily beget humiliation. Across Africa, relationships of dependency have been grounded in cultural norms and obligations that upheld the dignity of both parties. Becoming “wealth‐of‐others” could be a desired outcome under certain conditions, as it could facilitate access to social and material benefits (cf. Ferguson 2015; Guyer 1995; Kopytoff and Miers 1977). These were, however, communities where social hierarchies were naturalized, in contrast to Alexandra, where residents only foresaw humiliation as ensuing from entering into such relationships locally. Yet the rejection of dependency had a spatial dimension in Alexandra; dependency was easier if it was exogamic.

Together with the expansion of financial institutions into poorer residential areas, the growth of welfare policies in South Africa has amplified the significance of proximity for understanding dependency (Bähre 2020; Ferguson 2015). James Ferguson (2015, 159–64) has speculated what this entails for notions of dependency and social personhood, particularly if welfare policies are modeled on traditional forms of wealth‐in‐people. Taking men as potential future recipients of a Basic Income Grant, he wondered if impersonal cash transfers might be experienced as humiliating, making them passive recipients, stripped of the reciprocal obligations and social membership associated with traditional dependence. Yet, this perspective does not consider how citizens can use the reconfiguration of the state to reimagine notions of dependency/belonging‐to. A comparable process has been observed by Bähre (2020) in relation to how the growth of insurance companies in townships in Cape Town became an alterative to local saving clubs.

Bähre (2020, 162–63) discovered that funeral insurance eased neighborly relations, because the blame for potential fraud could be directed at the funeral company, thus alleviating rumors or suspicion about this being the work of someone in the community. Bähre recognizes the strategic element of choice, which gives a more nuanced perspective on neoliberal intersubjectivity, demonstrating how maintaining dignity, along with a commitment to specific values, underpins decisions. In his pivotal work on debt, David Graeber (2011, 382) remarked how modern state bureaucracy was designed to crush any space for imagining an alternative future. Nevertheless, some citizens have discovered how the state is indeed good to think with when imagining new alternatives for evading indebtedness and combating social inequality.

## Concluding remarks

While civil society provided invaluable help during the pandemic, issues arising from having civil society perform the responsibilities of the state became apparent. An op‐ed in the *Daily Maverick* by the Lockdown Diaries Project concerning food distribution in different parts of Cape Town recorded similar worries as in Alexandra regarding the selection process and potential fraud (Anciano et al. 2020b). Local actors charged with distribution in Cape Town also had to grapple with how to legitimize the selection of recipients. In Alexandra, the COVID‐19 committee's attempt to mimic the relation between the state and residents during the distribution seemed like a sensible approach, but because the organizers were all local faces, this rather spurred concerns about interpersonal indebtedness/humiliation. Prior to the national lockdown, relationships between residents in the township and the relationship to the state were distinguished on the basis of how they generated different notions about dignity.

Relations to kin or neighbors had to be carefully managed to avoid ending up indebted. Relationships with the government did not spark similar concerns. Agency is the key signifier for differentiating between these types of dependency. Whereas the former required active agency to preserve dignity in order to realize an ideal of equality between two parties, the latter allowed for a suspension of agency and acknowledgment of hierarchy without humiliation. During the distribution of the local parcels, the difference between these two types of dependency/indebtedness crumbled, because the local distribution was being perceived as an unholy hybrid of the two types.

The Lockdown Diaries welcomed the government's decision in May to temporarily expand the social grants programs, arguing that the state should assume responsibility for the population's survival (Anciano et al. 2020b). I share their opinion about the state being part of the solution. Despite the pernicious effects of the lockdown, people's trust in the government remained surprisingly robust. It gives food for political thought to ponder whether the spontaneous embrace of the share was a(n) (un)conscious political act. By resisting the local distribution, residents reasserted the government's imagined monopoly over welfare services. This lends credence to those arguing that South Africa is ready for more progressive policies in the future, such as the recently proposed Basic Income Grant (Mahlaka 2021).

## Notes

1 The photos would be uploaded to their official Facebook page.

2 SASSA distributes grants on behalf of the Department of Social Development.

3 When speaking English, my study participants used the term *dignity* or *dignified*.
